# pH-responsive nanozymes: multifunctional platforms for biomedical catalysis toward clinical translation

**DOI:** 10.3389/fbioe.2026.1765647

**Published:** 2026-02-06

**Authors:** Xiaofei Zhuang, Shanshan Gao, Yueqin Tao, Mujie Yuan, Zhiwu Han, Ying Wang

**Affiliations:** 1 Department of Pharmacy, The Affiliated Hospital of Qingdao University, Qingdao, Shandong, China; 2 Department of Stomatology, The Affiliated Hospital of Qingdao University, Qingdao, Shandong, China

**Keywords:** nanozymes, pH-responsive, biomedical, applications, clinical translation, enzyme mimics

## Abstract

As an emerging class of smart nanomaterials, pH-responsive nanozymes are capable of realizing dynamic regulation of catalytic activity according to microenvironmental acidity and alkalinity. The material system encompasses noble metals, metal oxides, metal sulfides, carbon-based nanozymes, and metal-organic frameworks. Through engineering strategies such as surface ligand modification, heterogeneous atom doping and core-shell structure design, these nanozymes can achieve precise response to complex biological microenvironments, and show unique catalytic properties in the lesion site with specific pH. In recent years, pH-responsive nanozymes have been applied in various biomedical fields, such as tumor therapy, antimicrobial, wound healing, and anti-inflammation, to enhance therapeutic efficacy through controlled activation and targeted drug delivery. However, they still face many challenges in clinical translation, such as *in vivo* stability, toxicity assessment and precise regulation of activity. This paper reviews the research progress of pH-responsive nanozyme therapeutic systems and discusses the potential and challenges of integrating them with other nanotechnologies and therapeutic modalities, aiming to provide a reference and outlook for promoting their wider application in clinical diseases.

## 1 Introduction

Nanozymes are a class of engineered nanomaterials capable of mimicking the catalytic activity of natural enzymes (Zhang et al., 2025). These nanomaterials catalyze specific reactions under physiological conditions, making them potential alternatives to natural enzymes in biomedicine (Zandieh and Liu, 2024). Nanozymes outperform conventional natural enzymes in various therapeutic and diagnostic applications due to their environmental responsiveness, cost-effectiveness, catalytic efficiency, physiological stability and ease of functionalization (Yang et al., 2021; Chen X et al., 2023). Nanozymes have evolved from basic enzyme mimics to sophisticated therapeutic agents. The history of nanozyme research began in 2007 with Gao et al. (2007), who observed enzyme-mimicking activities in iron oxide nanoparticles, marking a pivotal moment in the field. Batrakova et al. (2007) advanced the research by creating catalase-packaged nanozymes for selective antioxidant delivery in Parkinson’s disease models, reducing oxidative stress. In 2010, Rosenbaugh et al. (2010) developed CuZnSOD nanozymes, specifically a polyion complex formed by electrostatic binding of copper/zinc superoxide dismutase (CuZnSOD) protein to a synthetic poly(ethyleneimine)-poly(ethyleneglycol) block copolymer, which inhibited angiotensin II-induced oxidative stress without toxicity, showing potential for cardiovascular therapy. In 2012, Wang et al. (2012) engineered a nanozyme that mimicked the RNA-induced silencing complex, demonstrating potent antiviral activity against the hepatitis C virus. In 2017, Huo et al. (2017) introduced sequential catalytic nanomedicine, combining glucose oxidase and Fe_3_O_4_ nanoparticles to trigger tumor cell apoptosis, showing promise for targeted cancer therapy. A year later, Zhao et al. (2018) constructed poly(vinylpyrrolidone)-modified Prussian blue nanoparticles with good physiological stability and biosafety, providing a potential alternative treatment option for patients with inflammatory bowel disease. These groundbreaking studies open up new possibilities for future applications of nanozymes in the clinical field, promising more precise treatments.

**FIGURE 1:**
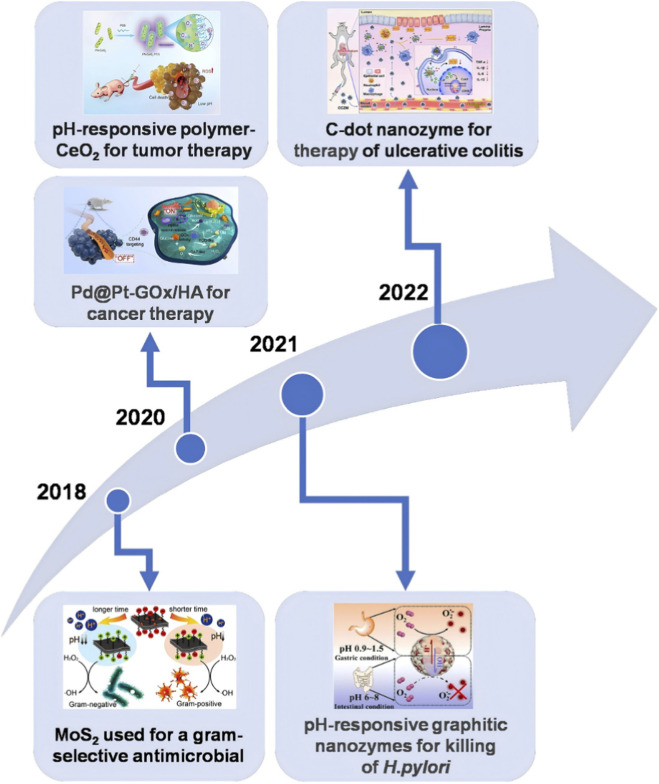
A timeline of some of the key discoveries in pH-responsive nanozymes to treat disease. Reproduced with permission from reference (Niu et al., 2018). Reproduced with permission from reference (Ming et al., 2020). Reproduced with permission from reference (Tian et al., 2020). Reproduced with permission from reference (Zhang L et al., 2021). Reproduced with permission from reference (Ma et al., 2022).

However, if nanozymes lack specificity and controlled activity, they can lead to non-specific interactions with biological components and unintended catalytic reactions, potentially increasing cytotoxicity and off-target effects (Singh et al., 2023). To address these challenges, researchers have developed stimuli-responsive nanozymes, which are engineered to alter their catalytic activities in response to specific external or internal stimuli—such as pH, temperature, light, magnetic fields, and the presence of specific biomolecules or chemical agents. In terms of response characteristics, compared with physical signals such as light and magnetism, which need to be accompanied by energy transfer process, the change of pH can trigger the instantaneous change of surface charge or conformation of nano-materials, thus achieving a fast and highly sensitive “on-off” effect, which is very important for biosensing and real-time diagnosis (Wang et al., 2018). In addition, the trigger mechanism of pH can effectively avoid non-specific activation and the risk of thermal damage caused by it (Wang Y. et al., 2024). As an environmental parameter with relatively stable physical and chemical properties, the degree of metabolic interference is lower than that of specific biomolecules, thus providing more reliable selectivity while ensuring the safety of treatment (Jiang et al., 2019). Since 2018, the evolutionary trajectory of pH-responsive nanozymes has shifted from basic antimicrobial uses to multifaceted therapeutic platforms. These advanced systems hold promise for addressing complex pathologies such as cancer, inflammatory diseases, and specific pathogen infections, highlighting their expanding clinical versatility in the era of precision medicine (Figure 1).

A pH-responsive nanozyme is defined as a nanomaterial with enzyme-mimetic activity that exhibits qualitative or quantitative switching of catalytic functions (e.g., activation/inactivation of specific enzyme-like activities, or conversion of catalytic types) in response to pH changes within physiologically relevant ranges, rather than merely exhibiting activity fluctuations due to general pH-dependent surface properties (Ren et al., 2022). This pH responsiveness may be due to the ionization states of functional groups on the surface of the nanozymes, which can change with pH, thereby altering their surface charge and catalytic properties (Chen X et al., 2023). In additional, substrate reactivity itself exhibits significant pH dependence that cannot be overlooked. For instance, under acidic conditions (pH 4-6), hydrogen peroxide (H_2_O_2_) predominantly exists as protonated H_3_O_2_^+^ species with enhanced electrophilicity that favors Fenton-like reactions with transition metal-based nanozymes, whereas at neutral pH (7.0–7.4), H_2_O_2_ deprotonates to form hydroperoxyl anions (HO_2_^−^) with different redox potentials and reaction kinetics that may inhibit certain catalytic pathways (Feng K. et al., 2024). As demonstrated by Ji et al. (2021), the apparent pH-responsiveness of a nanozyme system often represents the net effect of these intertwined factors rather than solely the nanomaterial’s intrinsic properties, highlighting the necessity for holistic characterization approaches in designing clinically relevant pH-responsive nanozymes.

The primary objective of this review is to provide a comprehensive analysis of pH-responsive nanozymes, focusing on their material types, fundamental design principles, functionalities, and biomedical applications. It aims to elucidate the potential of these nanozymes in enhancing targeted therapies, highlighting recent advancements and ongoing challenges. The review will cover various commonly material types, including metals, metal oxides, metal sulfide, carbon dot nanozyme and metal-organic frameworks and will delve into their design principles and explore their applications in clinical areas such as cancer therapeutics, antimicrobial action and wound healing. Finally, the review will discuss the challenges encountered in the research of pH-responsive nanozymes, proposing solutions to achieve precise control over nanozyme activity, and aim to advance the understanding and development of pH-responsive nanozymes for clinical applications.

## 2 Representative pH-responsive nanozymes

In recent years, various types of pH-responsive nanozymes have been developed and widely used in disease treatment and diagnosis, opening up new avenues in the biomedical field. This review will introduce some classical pH-responsive nanozymes including noble metal, metal oxide, metal sulfide and metal-organic frameworks (MOFs) nanozymes and C-dot nanozyme etc. Each material type offers unique properties and mechanisms that enhance pH responsiveness (Table 1). The following sections will explore each of these material types in detail, discussing their properties and specific applications in biomedical settings, thereby providing a comprehensive understanding of their role in enhancing targeted therapies and diagnostics.

**TABLE 1:** Characterization parameters of representative pH-responsive nanozymes.

Nanozymes	Size [nm]	Optimum pH value	Catalysis	Km (mM)	Vmax (μM·s^–1^)	References
PtCo	3	1.0	OXD	0.0561	0.034	Deng et al. (2023)
Dex-IONP	7.3	4.5	POD	0.0414	-	Huang et al. (2021)
CuCH-NCs	18.8	5.5	POD	1.55	1.278	Li et al. (2023b)
CoSnO_3_	3.6	4.5–6.5	POD,CAT	POD:1.2 CAT:0.1	POD:0.14 CAT:3.5	Hu et al. (2024)
MnO_2_-Au	100	3.5	POD	0.00161	1.23	Yu et al. (2023)
FeMOF	-	5.5	POD	10.22	0.09287	Liu C. et al. (2024)
COF_FePc_ NS	-	3.5–5.5	POD	3.036	1.35	Rong et al. (2024)
Carbon-dot	12	4.55	POD	0.028	36,378	Wang et al. (2020)
Zr/Ce-MOFs/DOX/MnO_2_	280	5.8	POD	1.21	0.00368	Song et al. (2023)
CuFeOx	7	5.4	POD	0.242	0.0099	Zhao et al. (2024)
Au@Pt	25	4	POD	6.794	13.262	Fu et al. (2021)
PFOB@PLGA@Pt	4	3–6	POD	26.96	1.72	Zhou et al. (2023)

### 2.1 Noble metal

The catalytic mechanism of noble metal nanozymes under different pH conditions is complex and not fully understood. This complexity arises from the interaction of several factors influenced by pH changes. This pH-dependent mechanism has a key role in the optimization of performance for medical applications, and its influence is mainly attributed to changes in surface charge, and electron transfer processes. Noble metal-based nanozymes have significant surface charge effects. The pH value of the environment can alter the surface charge of nanozymes. The change in surface charge can determine the electrostatic interaction between nanozymes and substrates, potentially enhancing or inhibiting their catalytic activity. The surface charge of precious metal nanoparticles, represented by gold (Au) and platinum, is highly responsive to changes in pH (Li et al., 2015). pH fluctuations change the ionization state of surface atoms, which modulates the adsorption behavior of charged substrates and intermediates, and ultimately affects the catalytic efficiency (Alizadeh and Salimi, 2021; Liu Q. et al., 2021). In addition, the catalytic mechanism of noble metal nanozymes usually involves a pH-dependent electron transfer process. The change in pH value can alter the electronic structure of nanozymes, thereby affecting their ability to promote electron transfer and thus affecting their catalytic efficiency. The ambient pH directly affects the reaction kinetics and product distribution by regulating the electron transfer efficiency (Chen J. et al., 2021; Zandieh and Liu, 2021).

Au nanoparticles (Au NPs) Primarily exhibit oxidase/peroxidase-like activities with lower efficiency compared to Pt. For example, Au NPs showed weaker tetramethylbenzidine (TMB) oxidation than Au@Pt composites (Fu et al., 2021). Compared with Pt NPs, Au NPs has a relatively low affinity for H_2_O_2_ and a moderate catalytic rate. However, Au NPs offer excellent biocompatibility and surface functionalization capabilities. Their catalytic activity can be significantly enhanced under acidic conditions, making them suitable for targeting acidic microenvironments such as tumor tissues or inflammatory sites. Pt’s electron-rich surface and porous nanostructure facilitate rapid electron transfer, achieving a 10-fold higher peroxidase-like activity than Au NPs. For example, Au@Pt NPs demonstrated a detection limit of 11 ng·mL^−1^ for SARS-CoV-2 spike protein, outperforming horseradish peroxidase (HRP)-based assays (Fu et al., 2021). The porous structure of Au@Pt NPs creates numerous active sites, resulting in catalytic efficiency that significantly surpasses both monometallic counterparts and even natural HRP. The synergistic effect between gold and platinum enhances pH adaptability, with these nanozymes maintaining high activity across a wider pH range than either metal alone.

### 2.2 Metal oxide

The concept of nanozymes was first introduced in 2007 when ferromagnetic Fe_3_O_4_ nanoparticles were reported to exhibit peroxidase-like activity (Gao et al., 2007). This groundbreaking research sparked further exploration into a variety of metal-based nanoparticles, particularly metal oxides, which share similar attributes with iron oxide nanoparticles. Due to their exceptional durability, hardness, and chemical resistance, metal oxides are often preferred over noble metals, primarily for their enhanced stability under various conditions (Liu Q. et al., 2021). Cerium oxide and iron oxide nanoparticles are notable examples. Cerium oxide nanoparticles exhibit unique redox properties, making them effective in catalysis applications. Iron oxide nanoparticles, known for their magnetic properties and biocompatibility, are widely used in medical imaging, drug delivery, and hyperthermia treatments. These attributes make both cerium oxide and iron oxide nanoparticles valuable in diverse biomedical applications.

#### 2.2.1 Cerium oxide

Cerium oxide nanoparticles (Ce NPs) have garnered significant attention in biomedical applications due to their unique redox properties and enzyme-mimetic activities (Xu and Qu, 2014). Nanoceria’s activity is uniquely stable across a broad range of pH levels due to the regenerative nature of their surface states (Singh, 2016). Cerium oxide nanozymes can interchange between Ce^3+^ and Ce^4+^ oxidation states based on the surrounding pH conditions (Feng et al., n.d.). In neutral pH environments, such as those found in normal cells, Ce NPs exhibit antioxidant properties, protecting cells from oxidative stress. However, in the acidic pH typical of tumor microenvironments, Ce NPs can act as pro-oxidants, generating reactive oxygen species (ROS) to induce cytotoxic effects on cancer cells. This pH-dependent behavior is attributed to the reversible Ce^3+^↔Ce^4+^ conversion and oxygen vacancy formation.

Cerium oxide nanoparticles exhibit oxidase-like activity, which can be significantly enhanced by surface modifications such as fluoride capping. This modification increases the turnover number and catalytic efficiency, making nanoceria more comparable to natural oxidases. The enhancement is attributed to changes in surface charge and improved electron transfer, which are crucial for catalytic activity at different pH levels (Liu et al., 2016). The peroxidase-like activity of cerium oxide is also pH-dependent. At acidic pH, cerium oxide nanozymes can catalyze the oxidation of substrates like 3,3′,5,5′-TMB using H_2_O_2_ as an oxidant. This activity can be tuned at neutral pH using adenosine triphosphate, which stabilizes the oxidation product and enhances catalytic performance (Benazir et al., 2020). Cerium oxide nanoparticles mimic superoxide dismutase (SOD) and catalase (CAT) activities, which are crucial for decomposing ROS like superoxide radicals and H_2_O_2_. The SOD-like activity is influenced by the Ce^3+^ surface area concentration, with smaller particles and higher Ce^3+^ fractions showing enhanced activity. The CAT-like activity, on the other hand, is modulated by the adsorption of H_2_O_2_ on the nanoparticle surface, which varies with pH. In chemodynamic therapy, cerium oxide nanozymes can regulate H_2_O_2_ homeostasis by enhancing SOD-like and peroxidase-like activities while inhibiting catalase-like activity. This regulation is crucial for generating toxic hydroxyl radicals in tumor regions, which are effective in inducing tumor apoptosis (Yang P. et al., 2022).

#### 2.2.2 Iron oxide

Iron oxide nanozymes are a class of magnetic nanoparticles that exhibit enzyme-like activities influenced by pH variations, making them highly valuable for various biomedical applications (Yang P. et al., 2022). Iron oxide nanoparticles exhibit pH-dependent enzyme-like activities, particularly peroxidase-like activity in acidic conditions and catalase-like activity in neutral environments (Chen et al., 2012). This pH-dependent behavior is attributed to the surface properties and redox state of iron atoms on the nanoparticle surface (Wan et al., 2022). The peroxidase-like activity involves the production of complexed hydroxyl radicals and high-valent FeO species, rather than just free hydroxyl radicals as previously thought (Wan et al., 2022). The structural and morphological properties of iron oxide nanoparticles, which are affected by pH, play a significant role in their catalytic efficiency. For example, the protonation or deprotonation of functional groups on the nanoparticle surface at specific pH levels can alter their electronic properties and reactivity, enhancing catalytic activity in organic transformations (Adwoa, 2024). In cancer therapy, the pH-dependent catalytic activity of iron oxide nanozymes is exploited to generate ROS in the acidic tumor microenvironment, enhancing their therapeutic efficacy (Sutrisno et al., 2020).

### 2.3 Metal sulfide

Metal sulfides exhibit enzyme-like activities that are influenced by environmental pH. The catalytic properties can be adjusted by altering the pH, which affects the nanozyme’s interaction with substrates and its overall activity. This adaptability is crucial for applications in varying physiological conditions (Zhang D. et al., 2024). Copper sulfide (CuS) nanozymes are notable for their enzyme-like catalytic activities, stability, and cost-effectiveness, making them a prominent focus in the biomedical field. These nanozymes mimic the functions of natural enzymes, such as peroxidase, catalase, and oxidase, and their activity can be modulated by altering the pH, which is crucial for their application in various biomedical fields (Zhang Y et al., 2021). The pH-dependent catalytic behavior of CuS nanozymes is primarily due to the structural and electronic changes that occur at different pH levels, affecting their interaction with substrates and the efficiency of catalytic reactions (Zhu et al., 2019). These activities are influenced by the oxygen vacancies in the CuS structure, which play a crucial role in their catalytic mechanisms (Xi et al., 2019). CuS nanozymes demonstrate significant peroxidase-like activity, particularly in acidic environments. This activity is attributed to the oxygen vacancies in the CuS structure, which facilitate the breakdown of H_2_O_2_ into ROS (Zhang Y et al., 2021). The peroxidase activity is optimal at lower pH values, such as pH 4, where the dissociation of H-O bonds in H_2_O_2_ is enhanced, leading to effective catalysis in Fenton-like reactions (Zhang Y et al., 2021). Catalase-like activity of CuS nanozymes is prominent in neutral to slightly acidic conditions. This activity involves the decomposition of H_2_O_2_ into water and oxygen, which is crucial for reducing oxidative stress in biological systems (Liu J. et al., 2024). The catalase activity is particularly beneficial in tumor microenvironments, where it can alleviate hypoxia by increasing oxygen levels, thus enhancing the efficacy of cancer therapies (Liu J. et al., 2024). CuS nanozymes also exhibit oxidase-like activity, which is more effective in slightly acidic to neutral pH conditions. This activity involves the oxidation of substrates in the presence of oxygen, contributing to the regulation of intracellular ROS levels (Feng K. et al., 2024). The oxidase activity is less effective in highly acidic or alkaline conditions, indicating a narrow pH range for optimal performance.

### 2.4 Metal–organic frameworks

MOFs are a class of porous materials constructed from metal ions or clusters coordinated to organic linkers (Lu et al., 2014). These structures are distinguished by their highly ordered, lattice-like frameworks, which can be tailored for specific applications due to their customizable pore sizes and functional surfaces (Luo et al., 2019). MOFs have emerged as a promising platform for developing pH-dependent nanozymes in the biomedical field. MOFs can mimic the catalytic activity of natural enzymes, and their efficiency and selectivity can be tailored by modifying the metal nodes and organic linkers (Islamoglu et al., 2017). This property allows for the design of MOFs with specific catalytic functions that can be activated under certain pH conditions found in tumor sites, enhancing the efficacy of the treatment. Liu C. et al. (2024) demonstrated that FeMOF can exhibit Fenton-like catalytic activity under acidic conditions, making it a promising candidate for cancer therapy. This unique property arises from the ability of FeMOF to facilitate the decomposition of endogenous H_2_O_2_ into highly reactive hydroxyl radicals in an acidic environment. The Fenton-like activity of FeMOF is highly pH-dependent, with optimal activity occurring in acidic environments. This is due to the fact that acidic conditions stabilize Fe^2+^ ions and promote the formation of hydroxyl radicals. The acidic tumor microenvironment precisely provides an ideal environment for the activation of the Fenton-like catalytic activity of FeMOF, which results in the efficient generation of reactive oxygen species in tumor cells, and thus induces oxidative stress injury in tumor cells.

### 2.5 Carbon dots nanozymes

Carbon dots (C-dots) are highly promising for biomedical applications such as drug delivery and bioimaging due to their minimal cytotoxicity and excellent biocompatibility (Rani et al., 2022; Abbas et al., 2024; Ullah et al., 2024). However, their utility can be constrained by a lower catalytic efficiency compared to some noble metal nanozymes, which may limit their effectiveness in applications requiring high catalytic activity (Kong and Zhou, 2024). Conversely, while noble metal and metal oxide nanozymes often exhibit superior catalytic performance, their higher toxicity and lower biocompatibility present significant limitations for their use in biomedical contexts (Ansari et al., 2024). This creates a fundamental trade-off between C-dots, which offer superior safety profiles, and metal-based nanozymes, which provide higher catalytic power, necessitating careful material selection based on the specific requirements of the intended application.

The acidic tumor microenvironment (approximately pH 6.0) is a critical triggering condition for the peroxidase (POD)-like activity of C-dot nanozymes. In acidic conditions, they exhibit POD-like activity by converting H_2_O_2_ into highly toxic hydroxyl radicals (Gao et al., 2023). This oxidative process can damage cell membranes, DNA, and proteins, ultimately causing cell death. Wang et al. (2020) demonstrated that C-dots could be incorporated into pH-sensitive supramolecular micelles for targeted cancer therapy. In the acidic tumor microenvironment, C-dot nanozymes exhibit peroxidase-like activity, converting H_2_O_2_ into highly toxic hydroxyl radicals, which directly disrupt the structural integrity of tumor cells to achieve efficient catalytic therapy. Conversely, in a physiologically neutral environment (pH 7.4), these nanozymes demonstrate catalase-like activity, decomposing H_2_O_2_ into non-toxic water and oxygen, thereby effectively reducing oxidative damage to normal tissues and enhancing biosafety. The pH responsiveness of C-dot nanozymes is a core mechanism enabling tumor-targeted catalytic therapy. This responsiveness is characterized by the switch between peroxidase-like activity under acidic conditions and catalase-like activity under neutral conditions, thereby balancing therapeutic efficacy with biocompatibility.

## 3 Fundamentals of pH-responsive nanozyme design

**FIGURE 2:**
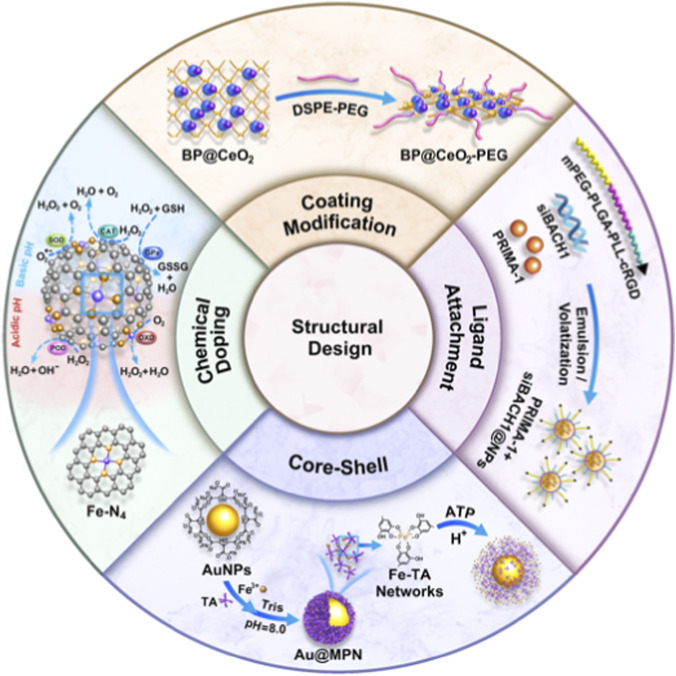
The summary diagram shows the basic structure and form of pH responsive nanozyme design, including surface modification techniques, chemical doping, and core-shell structure.

The design of pH-responsive nanozymes is a sophisticated approach that leverages the unique properties of nanozymes to respond to specific pH environments, enhancing their functionality in biomedical applications. These nanozymes mimic natural enzymes and are engineered to perform catalytic activities that are activated or enhanced in response to pH changes, making them particularly useful in targeted therapies and diagnostics. The fundamental aspects of pH-responsive nanozyme design include surface modification technology, chemical composition and doping control, and core-shell structures to ensure precise activation in desired environments (Figure 2).

### 3.1 Surface modification techniques

The principles of surface modification, particularly through ligand attachment and polymer coating, are foundational to tailoring the interface between nanozymes and their surroundings. Surface modification and functionalization are integral processes in optimizing nanozyme performance by altering their surface characteristics through the attachment of functional groups or molecules (Cao-Milán et al., 2017; Liu and Liu, 2017). This enhancement not only boosts the responsiveness, stability, and biocompatibility of nanozymes but also facilitates targeted activation, thereby improving their efficacy in therapeutic and diagnostic applications (Kumar et al., 2023). In the context of pH-responsive nanozymes, techniques like coating design and ligand attachment play key roles in enhancing stability, biocompatibility, targeting precision, and therapeutic effectiveness, each method offering unique advantages and mechanisms that cater to specific biomedical applications.

#### 3.1.1 Ligand attachment

Ligand attachment refers to the process of covalently bonding or associating functional molecules (such as proteins, peptides, or small molecules) to the surface of nanozymes, thereby modulating their biocompatibility, targeting ability, and catalytic performance (Nobs et al., 2004; Xia et al., 2017). For pH-responsive nanozymes, the ligand attachment structure serves as a critical functional switch. This is due to the difficulty of addressing the three core challenges of biocompatibility, stability, and targeting solely through the inherent pH responsiveness of the nanozymes themselves. In contrast, the introduction of specific ligand modifications can significantly optimize system performance through multiple synergistic mechanisms. Yang L. et al. (2022) utilized bovine serum albumin (BSA) as a ligand to modify CeO_2_ nanoparticles, successfully constructing the BSA@CeO_2_/Fe^2+^ nanozyme. The coupling of BSA is primarily based on affinity interactions, whereby its unique spatial structure and flexible molecular chains can closely bind to the surface of CeO_2_ through non-covalent forces such as electrostatic interactions and hydrophobic effects. The modification of BSA not only preserves the inherent pH-responsive characteristics of the nanozyme but also enhances its functionality through microenvironmental regulation. In the acidic tumor microenvironment (pH 6.5), the weakly acidic groups of BSA (such as carboxyl groups) serve a local buffering role, facilitating the redox cycling of Ce^3+^/Ce^4+^ and accelerating the conversion of superoxide anion to H_2_O_2_. This, in turn, leads to the efficient generation of highly toxic hydroxyl radicals via the Fe^2+^-mediated Fenton reaction. Conversely, in the neutral environment of normal tissues (pH 7.4), the amino groups of BSA tend to stabilize the Ce^4+^ state, thereby enhancing the nanozyme’s capacity to scavenge ROS and effectively mitigating oxidative damage to normal cells.

#### 3.1.2 Coating modification

**FIGURE 3:**
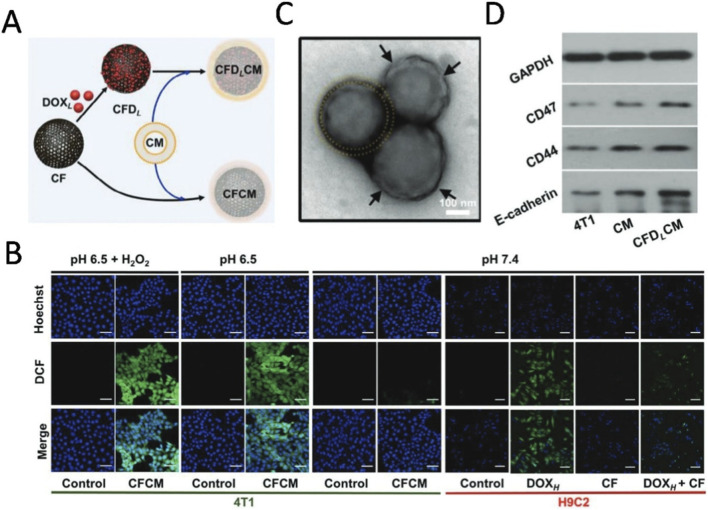
**(A)** Schematic representation of 4T1 tumor cell membranes fused with DOX to COF-derived carbon nanozymes. **(B)** Intracellular ROS fluorescence signal plots of 4T1 cells (50 μm) and H9C2 cells (100 μm) from different treatment groups under normal physiological conditions (pH 7.4) versus tumor acidic conditions. Green fluorescence indicates the signal of ROS detections. **(C)** 4T1 TEM image of tumor cell membrane-encapsulated COF-derived carbon nanozymes. **(D)** Western blot images of membrane proteins (E-calcineurin, CD44 and CD47) from 4T1 cells, 4T1 cell membranes and CFDLCM. Reproduced with permission from reference (Lv et al., 2024).

Certain pH-responsive nanozyme carriers are covered with a cellular biofilm coating. The biofilm has a tumor cell-specific targeting ligand, such as an antibody or peptide chain, and the nanozyme carrier can be precisely recognized and anchored to the surface of the tumor cell, enabling precise delivery of the chemotherapeutic agent. Lv et al. (2024) developed a carbon nanozyme derived from a covalent organic framework (COF) that exhibits multifunctional enzyme mimicry activity at different pH conditions (Figure 3A). Research indicates that this nanozyme can effectively generate ROS under acidic conditions, inducing oxidative stress to achieve tumor ablation. Conversely, under normal physiological pH, its ROS production capacity significantly diminishes, exhibiting antioxidant enzyme activities that clear ROS and protect cardiomyocytes from drug-induced oxidative damage (Figure 3B). In addition, through its porous structure and high specific surface area, the nanozyme realizes the payload of the anticancer drug doxorubicin (DOX), and utilizes tumor cell membrane coating technology to improve the stability and targeted delivery efficiency of the drug, further enhancing the therapeutic effect and reducing non-targeted accumulation and side effects. The researchers uniformly coated the surface of DOX-loaded CF nanoparticles with 4T1 tumor cell membranes, which facilitates the controlled release of the drug through membrane rupture or fusion while retaining membrane-specific proteins such as E-cadherin, CD44, and CD47 (Figures 3C,D). These proteins are crucial for cancer cell targeting, homotypic adhesion, and immune evasion. These properties make the nanozyme a highly effective pH-responsive chemotherapy carrier, providing a new strategy for cancer treatment.

### 3.2 Chemical composition and doping

In 2018, Fan et al. (2018) pioneered the development of nitrogen-doped porous carbon nanospheres (N-PCNSs) exhibiting enzyme-mimetic activities. These nanozymes demonstrated the ability to synergistically regulate ROS levels within lysosomes, thereby inducing tumor cell death and highlighting the significant biomedical potential of nitrogen-doped carbon nanomaterials. This seminal work established a robust paradigm for integrating metal or non-metal dopants into nanozyme architectures, a design strategy that has since garnered widespread recognition within the academic community. This process, known as doping, can generate new catalytic sites or modify existing ones, thereby improving catalytic activity, stability, and pH responsiveness (Xi et al., 2021). Liu J. et al. (2021) introduced nitrogen atoms into titanium dioxide nanoparticles (TiO_2_NPs) and found that it significantly increased the peroxidase activity of titanium-based nanozymes. Specifically, the degree of nitrogen doping was proportional to the POD activity. This improvement was attributed to the fact that nitrogen is weaker electronegative than oxygen, thus creating a more favorable environment for the peroxidase reaction. It was found that the cascade catalytic activity of nitrogen-doped nanozymes was significantly enhanced under weakly acidic conditions in tumors, leading to a more efficient production of ROS in the tumor microenvironment, enabling them to effectively kill tumor cells.

### 3.3 Core-shell structure

Core-shell structures in pH-responsive nanozymes represent a sophisticated approach to enhancing the functionality, stability, and specificity of these catalytic nanomaterials (Joo et al., 2014). A core-shell structure involves a central core material surrounded by a shell layer. The core provides the catalytic activity, while the shell enhances stability, biocompatibility, and controlled release properties (Zhang and Wang, 2020). The catalytic activity of pH-responsive nanozymes is contingent upon pH modulation; however, this is predicated on the ability of the nanozymes to survive and precisely target in certain harsh physiological environments, such as gastric acid. The core-shell structure addresses the challenges of stability, selectivity, and biocompatibility that cannot be simultaneously achieved by a single type of nanomaterial through the physical protection, chemical regulation, and functional integration provided by the shell. Consequently, the core-shell architecture represents a critical design element for the clinical translation of these nanozymes. The pH-responsive graphite nanozyme (PtCo@G) designed by Zhang et al. features a core-shell structure, wherein the PtCo nanocrystal core is encapsulated within a graphite shell (Zhang L et al., 2021). This structural configuration is pivotal for the realization of its functional capabilities. The oxidase activity of this nanozyme exhibits a stringent pH dependence, being activated solely under acidic conditions to generate ROS, while being inhibited in the neutral intestinal environment, thereby preventing damage to the symbiotic microbiota. Notably, the graphite shell plays a crucial role in modulating the dissociation and adsorption behavior of O_2_ on the surface of PtCo, thereby deeply influencing the pH-responsive mechanism: it facilitates O_2_ adsorption under acidic conditions to drive ROS production, while suppressing this process in neutral conditions. Furthermore, the graphite shell not only provides resistance against corrosion in extreme acidic environments to protect the catalytic core but also significantly enhances biocompatibility and reduces off-target toxicity.

## 4 Biomedical applications of pH-responsive nanozymes

pH-responsive nanozymes have emerged as a promising tool in biomedical applications due to their unique properties that offer advantages over natural enzymes (Huang et al., 2019). These artificial enzymes, constructed from nanomaterials, exhibit enzyme-mimetic activities and can be engineered to perform specific catalytic functions under varying pH conditions. Their versatility and adaptability make them suitable for a range of therapeutic interventions, offering innovative solutions to complex medical challenges. This section explores their applications in diseases such as cancer treatment, wound healing, antimicrobial and anti-inflammatory, highlighting the relevant mechanisms and benefits of these innovative therapeutic tools (Table 2).

**TABLE 2:** Summarize representative pH-responsive nanozymes.

Nanozymes	Mechanism	Design method	Application	References
Pd	pH-dependent generation of hydroxyl radicals	Surface modification	Lung bacteria	Jin et al. (2023)
PtCo	ROS generated in acidic attacks bacterial membrane	Core-shell structure	*H. pylori*	Zhang L et al. (2021)
Dex-IONP	H_2_O_2_ generated by GOx is catalyzed to ROS	Enzyme surface coating	Oral bacteria	Huang et al. (2021)
BiO_2_	Sterilization under weak acid conditions; Anti-inflammatory under neutral or alkaline conditions	Single layer two dimensional	Bacterial sepsis	Liu F et al. (2024)
ZnO_2_@Ag	Generate oxygen under alkaline or neutral conditions and hydroxyl radicals under acidic conditions	Composite nano-frame	Anti-Bacterial	Jannesari et al. (2023)
Fe_3_O_4_	ATP forms a complex with Fe^2+^, activating the generation of hydroxyl radicals from H_2_O_2_	Enzyme surface coating	Anti-Bacterial	Vallabani et al. (2020)
AuNP	Under gastric acid, catalyzing the generation of ROS to attack bacterial cell membranes, while it is inactive under neutral conditions	Core-shell structure	*H. pylori*	Yan et al. (2021)
Cu_2_MoS_4_	Under acidic, ROS is killing bacteria and polarizing macrophages; under neutral, ROS is eliminated, inhibiting the polarization of M1 macrophages	Uniform Hollow structure	Anti-Bacterial	Yang et al. (2023)
FeAu	Activated under acidic, catalyzing ROS	MOF	Rectal cancer	Cao et al. (2024)
Au NPs @Mn@Cu	Under pH 6.0, Mn^2+^ is protonated. Mn^2+^ exhibits POD-like activity, catalyzing the decomposition of H_2_O_2_ to produce O_2_	Surface modification	Anti-tumor	Jia et al. (2024)
CuMn	Under acidic, catalyze singlet oxygen from H_2_O_2_, disrupting HSPs and P-gp in tumor cells	Two-dimensional N-doped C-nanosheets	Drug-resistant tumors	Li et al. (2023a)
CeO_2_	GOx consumes glucose to produce H_2_O_2_ and gluconic acid, lowering the pH and enhancing the peroxidase activity, photothermal and enhanced catalytic therapy	Core-shell structure	Anti-tumor	Shi et al. (2023)
FeMOF	siRNA silences MCT4, blocking lactate efflux and lowering intracellular pH; FeMOF catalyzes the generation of ROS from H_2_O_2_, attacking mitochondria	Core-shell structure	Anti-tumor	Liu C et al. (2024)
Pd@Pt	The HA layer is degraded, GOx catalyzes the oxidation of glucose to produce H_2_O_2_, and Pd@Pt catalyzes the generation of ROS	Core-shell structure	Anti-tumor	Ming et al. (2020)
BSA@CeO/Fe^2+^	Under pH = 6.5, the Fe^2+^ catalyzes the production of ROS to kill tumor cells; under pH = 7.4, the CeO catalyzes the removal of ROS to protect normal cells	Fe^2+^ doped composite structure	Anti-tumor	Yang L et al. (2022)

### 4.1 Cancer therapy

Traditional cancer therapies often demonstrate limited efficacy and can lead to severe side effects (Sulaiman et al., 2025). Tumor cells exhibit the Warburg effect, a phenomenon in which they rely primarily on glycolysis for energy production even in the presence of oxygen. Consequently, they secrete large amounts of lactate, lowering the extracellular pH to approximately 6.5–6.8, whereas normal tissue maintains a pH of about 7.4 (De La Cruz-López et al., 2019). Common nanozymes, if lacking pH responsiveness, may be prematurely activated in normal tissues, resulting in nonspecific toxicity; alternatively, they may exhibit insufficient activity within the tumor microenvironment due to unfavorable pH conditions. pH-responsive nanozymes achieve “on-demand activation” by sensing the acidic characteristics of the tumor microenvironment. They remain stable under normal physiological pH (7.4) and release active components and initiate catalytic reactions exclusively in the acidic tumor environment, thereby significantly enhancing tumor selectivity and reducing damage to normal tissues (Liu et al., 2025). Next, we will deeply explore the application of pH-responsive nanozymes in chemodynamic therapy, photothermal therapy and photodynamic therapy, and tumor microenvironment regulation, and reveal how they use innovative mechanisms to provide cancer treatment opens up new avenues.

#### 4.1.1 Synergistic therapy through combined catalytic activities

Existing nanozymes suffer from a lack of environmental adaptability and an inability to maintain redox balance, which may lead to side effects in physiological environments under a single activity (Singh, 2024). Moreover, they struggle to mimic natural antioxidant systems effectively in eliminating toxic intermediates. Wang et al. (2021) discovered that FePOs nanozyme can switch between three pH-dependent enzymatic activities (POD, SOD, and CAT-like activities) for the first time, achieving efficient catalytic killing in the tumor microenvironment while providing antioxidant protection in normal tissues. In the acidic tumor microenvironment, FePOs functions as a POD mimic, catalyzing the conversion of endogenous H_2_O_2_ into highly cytotoxic hydroxyl radicals through a Fenton-like reaction, effectively promoting apoptosis in cancer cells. However, in the neutral or slightly alkaline pH of normal tissues, FePOs exhibits SOD/CAT-like activity, scavenging both H_2_O_2_ and superoxide anions, thereby maintaining redox homeostasis and preventing oxidative stress-induced damage to normal cells. This pH-responsive switch, characterized by POD-like activity for tumor eradication combined with SOD and CAT-like activities for normal tissue protection, emulates the synergistic mechanisms of the natural antioxidant system. Consequently, this behavior effectively prevents oxidative damage to healthy tissues induced by both exogenous H_2_O_2_ and the nanomaterials themselves.

While chemodynamic therapy (CDT) relies on nanozymes to catalyze the conversion of H_2_O_2_ into cytotoxic hydroxyl radicals, conventional nanozymes are hindered by insufficient endogenous H_2_O_2_ levels for efficient radical generation and pose risks of off-target toxicity by decomposing H_2_O_2_ in normal tissues. The Pd@Pt-GOx/HA nanoreactor designed by Ming et al. (2020) enhances the tumor specificity of CDT through pH-responsive and cascade catalytic mechanisms. In this system, glucose oxidase (GOx) catalyzes the oxidation of glucose to generate H_2_O_2_, which serves as the substrate for the POD-like activity of Pd@Pt. This POD-like activity is significantly amplified within the acidic tumor microenvironment (pH 4.5–6.5) to efficiently catalyze the generation of hydroxyl radicals, whereas it remains suppressed under the neutral pH conditions of normal tissues. Consequently, the Pd@Pt-GOx/HA nanoreactor achieves efficient synergism between starvation therapy and CDT, presenting a novel therapeutic strategy characterized by reduced systemic toxicity and high tumor specificity.

#### 4.1.2 Synergistic therapy through multi-responsive mechanism

One of the notable characteristics of the TME is its weakly acidic nature (Peng et al., 2022). Although this acidic environment holds potential as a therapeutic target, conventional nanozymes often exhibit insufficient catalytic activity under acidic conditions. Furthermore, single therapeutic modalities, such as those relying solely on CDT, frequently struggle to achieve complete tumor eradication due to the lack of synergistic enhancement mechanisms (Wu et al., 2024). Combining pH response with other response mechanisms such as photothermal, researchers have developed multi-responsive nanozymes that can take advantage of the acidic microenvironment of tumors and respond to external stimuli such as near-infrared (NIR) light (Yuan et al., 2024). Tao et al. (2024) developed a pH-responsive multifunctional platform integrating Rh single-atom nanozyme (SA-Rh nanozyme) and photothermal therapy (PTT) for synergistic tumor treatment. In terms of enzymatic characteristics, their POD-like activity demonstrates significantly higher catalytic efficiency at pH 6.0 (simulating the tumor microenvironment) compared to pH 7.4 (normal tissue environment), effectively decomposing H_2_O_2_ to generate highly toxic hydroxyl radicals. Concurrently, their CAT-like activity can accelerate the decomposition of H_2_O_2_ into O_2_ under acidic conditions, which not only alleviates tumor hypoxia but also promotes the POD reaction cycle by replenishing substrates, thereby enhancing ROS generation through a substrate cycling mechanism. Regarding photothermal performance, SA-Rh achieves a photothermal conversion efficiency of up to 34.1% under 808 nm near-infrared light irradiation, enabling direct thermal ablation to kill tumor cells. More importantly, the localized high temperatures generated by PTT can significantly enhance the enzymatic activity of SA-Rh, inducing a burst-like production of ROS, thereby achieving a synergistic therapeutic effect of CDT and PTT. Generating oxygen can alleviate the hypoxic state in tumor tissue and further improve the therapeutic effect. pH-responsive SA-Rh nanozymes, through exploiting acidic activation catalysis, photothermal synergy, and the generation of multiple reactive oxygen species, have overcome the limitations of conventional nanozymes characterized by insufficient activity in the TME, limited substrate availability, and poor single therapeutic efficacy.

However, conventional nanozymes, due to their lack of the ability to autonomously regulate their microenvironment, face significant challenges in overcoming the dual bottlenecks of low H_2_O_2_ concentration and unsuitable pH levels within the TME (D and A, 2024). Shi and colleagues introduced a self-accelerating cascade catalytic nanoprobe, MPB@CeO_2_-GOx, which utilizes cerium dioxide to achieve dual functions in PTT and enhanced catalytic therapy (Shi et al., 2023). The nanoprobe consists of mesoporous Prussian blue (MPB), ultrasmall CeO_2_ and GOx. In the weakly acidic tumor microenvironment, CeO_2_ acts as a CAT mimetic enzyme to decompose endogenous H_2_O_2_ into O_2_ and water. The generated O_2_ promotes the consumption of glucose by GOx, further generating H_2_O_2_ and gluconic acid. The generated H_2_O_2_ and gluconic acid reduce the pH of the tumor microenvironment. The low pH further enhances the POD activity of CeO_2_ and increases the generation of hydroxyl radicals. MPB has a photothermal effect and can generate heat under light, significantly improving the POD activity of CeO_2_. MPB@CeO_2_-GOx employ a cascade strategy that couples endogenous H_2_O_2_ self-supply, intra-tumoral acidification, O_2_ recycling, and photothermal amplification to present a novel solution to the clinical challenges of tumor microenvironment suppression and insufficient therapeutic precision.

### 4.2 Wound healing

Diabetic wounds often exhibit an acidic microenvironment (pH ≈ 5.5–6.5) during the inflammatory phase due to bacterial metabolism and tissue necrosis, whereas in the later stages of healing, as inflammation subsides, the pH gradually returns to a weakly alkaline state (pH ≈ 7.4) (Fan et al., 2024). Nevertheless, within this context, multiple pathological obstacles, such as insufficient angiogenesis and bacterial biofilm formation, severely impede the healing process (Papadopoulou et al., 2023). Conventional nanozymes, characterized by static enzymatic activity and poor environmental adaptability, often lead to therapeutic contradictions—such as hindering antibacterial action during infection or failing to scavenge ROS during healing. In contrast, pH-responsive nanozymes address these limitations by intelligently sensing wound pH dynamics to switch between POD and CAT activities. The nanozyme hydrogel (Mo,Fe/Cu,I-Ag) designed by Li Q. et al. (2024) addresses the clinical challenges of diabetic wounds through pH-responsive cascade reactions and multi-enzyme synergistic effects. In an acidic environment, glucose oxidase catalyzes the oxidation of glucose, producing gluconic acid and hydrogen peroxide, which is then converted into hydroxyl radicals and superoxide anions by the gel’s peroxidase- and oxidase-like activities, effectively sterilizing the wound by destroying bacterial cell membranes. This process also directly reduces the local blood glucose concentration in the wound, thereby preventing the inhibitory effects of elevated glucose levels on the healing process. As the wound heals and the pH increases, the gel simulates superoxide dismutase activity to convert superoxide anions into oxygen and water, while also exhibiting CAT-like activity that decomposes hydrogen peroxide, reducing oxidative stress and alleviating hypoxia. This pH-responsive cascade not only improves the efficiency of the treatment, but also avoids the potential for secondary damage that can occur with conventional treatments.

**FIGURE 4:**
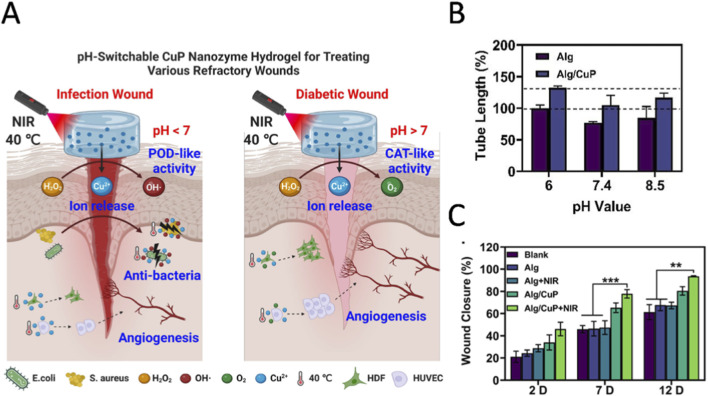
**(A)** Schematic diagram of the healing process of a multifunctional pH response cup nanozyme composite hydrogel (Alg/CuP) used for various refractory wounds. **(B)** The *in vitro* angiogenic potential of human umbilical vein endothelial cells (HUVECs) was assessed using tube formation assays conducted under different pH conditions. **(C)** Comparative analysis of wound closure rates in infected wounds subjected to various treatment modalities at 2, 7, and 12-Day intervals (Sample Size: n = 6). Reprinted (adapted) with permission from reference (Feng Y. et al., 2024).

Traditional wound dressings struggle to simultaneously achieve efficient antibacterial properties and tissue regeneration (angiogenesis and collagen deposition). However, pH-responsive nanozymes hold the potential to overcome this contradiction through a photothermal-enhanced synergistic repair mechanism. Feng Y. et al. (2024) developed a multifunctional wound dressing with a simpler preparation process that integrates traditional alginate (Alg) hydrogels with newly developed biodegradable copper hydrogen phosphate (CuP) nanozymes (Figure 4A). This composite dressing (Alg/CuP) exhibits near-infrared photothermal conversion capabilities, copper ion release properties, and pH-responsive peroxidase/catalase mimetic catalytic activity. When applied to diabetic wounds in an alkaline microenvironment, the Alg/CuP dressing converts H_2_O_2_ into dissolved O_2_ through its CAT-like activity, while continuously releasing copper ions to promote angiogenesis and accelerate wound healing (Figure 4B). In contrast, in cases where bacterial infection leads to an acidic wound environment, the catalytic performance of Alg/CuP shifts to POD-like activity, generating toxic hydroxyl radicals that work synergistically with copper ions to eliminate various bacteria and biofilms. This property enables dynamic adaptation to the pathological microenvironment of chronic wounds under varying pH conditions. Additionally, the mild thermal effect induced by near-infrared irradiation can enhance the catalytic activity and bioactivity of copper ions, thereby strengthening the healing process of infected and diabetic wounds (Figure 4C). The pH-responsive CuP nanozyme in this study addresses the core issues of the difficulty in balancing antibacterial effects and regeneration in clinical settings, thereby providing a novel solution for the treatment of chronic non-healing wounds.

### 4.3 Anti-bacterial action

The rapid emergence of antimicrobial resistance poses a critical challenge, rendering many traditional antibiotics ineffective against common bacterial infections (Muñoz-López et al., 2024; Zhou et al., 2024). Furthermore, conventional antibiotics frequently cause undesirable side effects and potential toxicity, which can limit their clinical application (Muñoz-López et al., 2024). As the efficacy of existing antibiotics continues to decline, there is an urgent need for the development of novel therapeutic agents (Ye et al., 2024; Zhang S. et al., 2024). In recent years, the pH-responsive nanozyme can respond under specific pH conditions, showing strong antibacterial activity, and can effectively treat infected parts. This section will focus on the new progress and innovative applications of pH-responsive nanozymes in antibacterial treatment, including their application in targeted treatment of *Helicobacter pylori* (H.pylori), oral infections and Methicillin-resistant *Staphylococcus aureus* (MRSA).

#### 4.3.1 Targeted therapy for *H. pylori*

**FIGURE 5:**
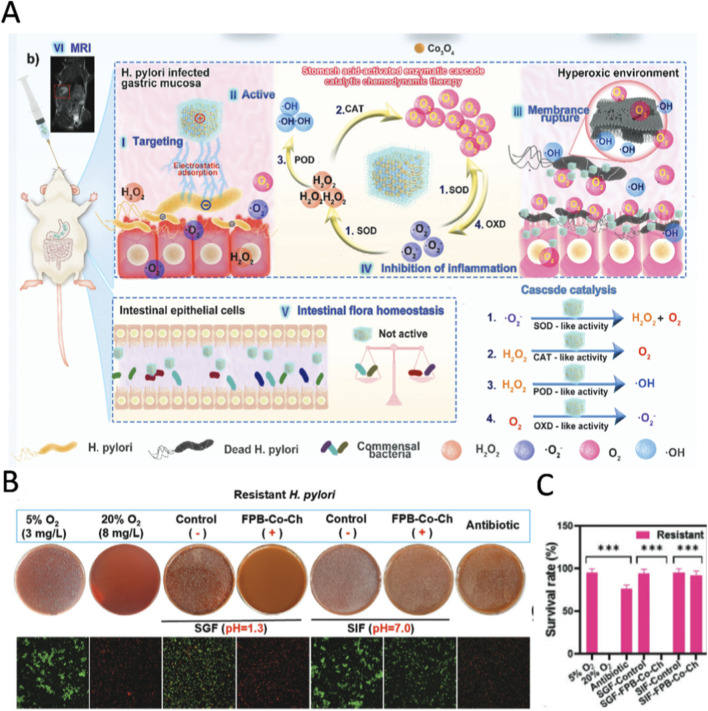
**(A)** Application scenarios of FPB cobalt carbon monoxide nanozyme complex. In the gastric environment, I) the deacetylated chitosan on the surface of the nanozyme achieves specific targeting of H.pylori, II) exerts antibacterial efficacy by activating multiple cascade nanozyme reactions (including oxidase OXD, superoxide dismutase SOD, peroxidase POD, catalase CAT), III) selectively kills H.pylori and destroys its cell membrane structure, and IV) exhibits significant anti-inflammatory effects. **(B)** Study on antibacterial activity against H.pylori *in vitro*. In simulated gastric fluid (SGF) or simulated intestinal fluid (SIF), drug-resistant H.pylori were treated with 5% O_2_, 20% O_2_, FPB-Co-ChNP, and antibiotics (amoxicillin, 0.04 mg·mL^−1^), respectively. Observation was conducted through plate dilution culture and confocal fluorescence staining techniques, where live bacteria showed green fluorescence and dead bacteria showed red fluorescence. **(C)** The survival rates of resistant group were quantitatively analyzed using plate counting method. Reproduced with permission from reference (Tong et al., 2024).

In the treatment of drug-resistant *H. pylori*, the precise targeting and environmental adaptability of pH-responsive nanozymes are of paramount importance, whereas conventional nanozymes often fall short of meeting these critical requirements. Firstly, the significant disparity between the highly acidic gastric environment (pH 1.3–2.0) and the neutral intestinal environment (pH 7.0) necessitates that therapeutic agents possess a specific activation mechanism. pH-responsive nanozymes are activated exclusively under acidic conditions, remaining inert in neutral environments, thereby achieving gastric-targeted therapy with no side effects in the intestine. Secondly, in addressing the challenges of antibiotic resistance arising from drug efflux and target mutations, pH-responsive nanozymes employ physicochemical mechanisms that do not rely on bacterial metabolic pathways for bactericidal activity, fundamentally circumventing the issue of resistance (Yan et al., 2021). Tong et al. (2024) developed a nanozyme (FPB-Co-ChNPs) composed of ferrocyanide and Co_3_O_4_ for targeted eradication of drug-resistant *H. pylori* infection (Figure 5A). The surface of the nanozyme is modified with chitosan, which enables it to have a targeted adhesion effect on bacteria. In the gastric acid environment, FPB-Co-ChNPs further increase intracellular oxygen concentration through SOD mimicking activity and CAT mimicking activity, making it impossible for microaerobic H.pylori to survive. Secondly, POD simulated activity uses H_2_O_2_ to produce hydroxyl radicals. Hydroxyl radicals have extremely strong oxidizing ability and can destroy the cell membrane and intracellular structure of bacteria, thereby achieving a bactericidal effect. Finally, oxidase simulated activity accelerates the above reaction, promotes the generation of hydroxyl radicals, and enhances the overall antibacterial effect. Conversely, neutral intestinal environment (pH 7.0) inhibits its enzymatic activity, thereby reducing O_2_ production and minimizing impact on the intestinal flora (Figure 5B). For drug-resistant H.pylori, this nanozyme activated in an acidic environment is more effective than the standard first-line triple therapy (Figure 5C).

However, this strategy also has limitations, as single target strategies may lead to non-specific adsorption of drugs on gastric mucosal epithelial cells, reducing selectivity and effectiveness towards target bacteria. To overcome these shortcomings, the researchers redesigned a dual-target strategy for the treatment of *H. pylori*. Deng et al. (2023) employed a novel dual-targeting strategy in conjunction with pH-responsive cascade catalysis design to develop the nanozyme PtCo@Graphene@Hemin-2 (L-arginine) (PtCo@G@H2A), aimed at effectively targeting and treating *H. pylori*. This nanozyme utilizes the receptor-targeting action of heme binding to the bacterial surface protein HugZ, combined with an environmental targeting mechanism triggered by the protonation of L-arginine in an acidic environment, achieving a targeting efficiency that is 850% higher than that of single-target approaches. Furthermore, to address antibiotic resistance, this design circumvents traditional metabolic pathways by employing a pH-responsive cascade catalytic reaction—initially catalyzing the generation of ROS from O_2_ via oxidase activity, which subsequently oxidizes L-arginine to produce nitric oxide, ultimately generating the highly oxidative ONOO^−^. This process results in bacterial membrane damage and DNA fragmentation, with an *in vitro* bactericidal rate reaching up to 86.9%. The PtCo@G@H2A in the paper further achieves the efficient and safe elimination of antibiotic-resistant H.pylori through dual-targeting and cascade catalysis, providing a novel strategy to overcome antibiotic resistance and facilitate drug delivery in extreme environments.

#### 4.3.2 The therapy of dental caries

**FIGURE 6:**
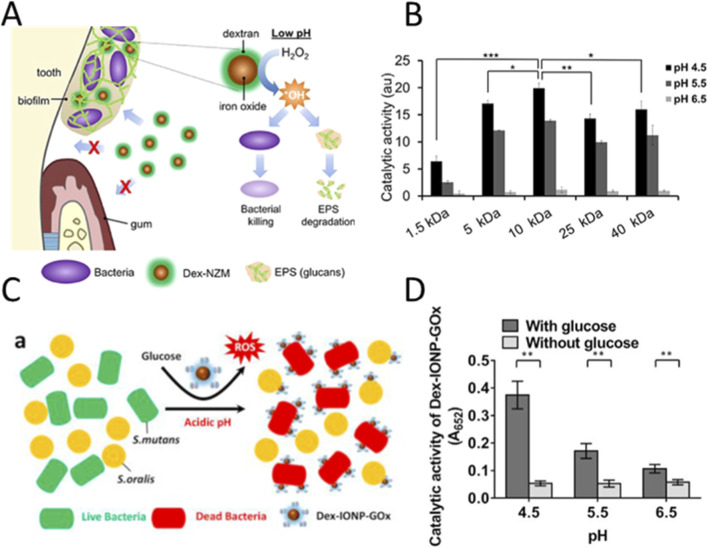
**(A)** The schematic diagram demonstrates the generation of hydroxyl radicals by dextran-coated iron oxide nanomaterials (Dex-NZM) via the Fenton reaction at low pH, which kills bacteria and degrades extracellular polysaccharides (EPS) against dental plaque. **(B)** Based on the TMB colorimetric method, the catalytic activities of the different Dex-NZM formulations were determined at three different pHs. Reproduced with permission from reference (Naha et al., 2019). **(C)** The schematic diagram depicts the selective binding and killing mechanism of the proposed Dex-IONP-GOx against *Streptococcus* pyogenes (S. mutans) and *Streptococcus* oralis (S. oralis). **(D)** The catalytic activity of Dex-IONP-GOx was evaluated at different pH and with or without glucose based on reactive oxygen species produced by the sequential reaction (n = 3). Reproduced with permission from reference (Huang et al., 2021).

Dental caries are primarily caused by biofilms, which are structured microbial communities that protect cariogenic bacteria, making them resistant to conventional treatments. The extracellular matrix of biofilms enhances adhesion and cohesion, creating a challenging environment for therapeutic agents to penetrate and act effectively (Liu et al., 2018). The localized acidic environment within biofilms, resulting from carbohydrate fermentation by bacteria, leads to tooth demineralization. This low pH environment is difficult to neutralize with traditional treatments, which often fail to selectively target and disrupt the biofilm without affecting the surrounding oral tissues (He et al., 2023; Wang et al., 2023). Nevertheless, this acidic microenvironment is a critical driver of dental caries and offers specific targets for therapeutic intervention. pH-responsive nanozymes exhibit enhanced catalytic activity under such acidic conditions, effectively converting H_2_O_2_ into hydroxyl radicals to disrupt biofilms and thereby suppress bacterial growth. Naha et al. (2019) designed a novel nanomaterial, dextran-coated iron oxide nanoparticles (Dex-NZM), for treating biofilms associated with dental caries (Figure 6A). The POD-like activity of Dex-NZM is significantly enhanced at acidic pH (such as 4.5), while exhibiting lower activity in neutral or alkaline environments (Figure 6B). This indicates that its catalytic activity is activated solely within the acidic microenvironment of dental caries biofilms, where it decomposes H_2_O_2_ to produce free radicals, thereby achieving localized bacterial eradication and the degradation of extracellular polysaccharide matrices, without significantly affecting the surrounding healthy oral tissues, such as gingival cells and the enamel coated by saliva. pH-responsive Dex-NZM addresses the issues of non-specific toxicity, poor stability, and inadequate clinical applicability associated with conventional nanozymes (such as uncoated NZM) through the activation by acidic microenvironments, targeted binding to biological membranes, enhanced stability, and improved biocompatibility, thereby offering an efficient and safe novel strategy for the treatment of dental caries.

Existing strategies are hampered by the dependence on exogenous H_2_O_2_, which requires a complex two-step administration (Nanoparticles + H_2_O_2_) that risks local cytotoxicity due to excessive peroxide concentrations (Dirersa et al., 2024). Furthermore, the non-specific nature of broad-spectrum agents like chlorhexidine results in collateral damage to the commensal microbiota, disrupting microbial diversity and driving the development of resistance (Bartsch et al., 2024). Huang et al. (2021) designed a dual-catalytic nanohybrid system to activate and precisely target bacterial pathogens in biofilm microenvironments (Figure 6C). The system consists of GOx covalently bonded to dextran-coated iron oxide nanoparticles (Dex-IONP) to form a Dex-IONP-GOx nanohybrid system. The nanohybrid system takes advantage of the high sugar and low pH conditions in the biofilm microenvironment to convert glucose in the biofilm into H_2_O_2_ through GOx, and then Dex-IONP only catalyze H_2_O_2_ to generate ROS under acidic conditions (pH 4.5) (Figure 6D). The dextran on the surface of Dex-IONP can specifically bind to the glucan-binding proteins on the surface of *streptococcus* pyogenes (such as GtfB), while the commensal bacterium *Streptococcus* oralis (*S. oralis*) lacks such proteins, resulting in the accumulation of the nanozyme on the surface of the pathogenic bacteria and the localized production of a higher concentration of ROS. The combination of an acidic environment-triggered mechanism and the selective binding to pathogenic bacteria ensures that ROS are generated exclusively at lesion sites. This targeted production prevents nonspecific damage to healthy tissues, such as the gingiva and oral mucosa, as well as symbiotic bacteria like S. oralis.

#### 4.3.3 Targeted therapy for MRSA

MRSA is a bacterium that is resistant to a wide range of antibiotics, largely due to its ability to form biofilms (Le and Otto, 2024). Biofilms are complex polysaccharide-protein complexes secreted by bacteria to evade antibiotics and the host immune system, and they frequently exhibit an acidic microenvironment (Ph 4.5–6.5) resulting from glycolytic metabolism (Deusenbery et al., 2023; Zhao et al., 2023). Conventional nanozymes, however, lack environmental responsiveness and cannot dynamically regulate their activity based on the lesion’s microenvironment. pH-responsive nanozymes can modulate their activity in response to environmental pH changes. This “intelligent switching” mechanism ensures the precise release of antibacterial agents at pathological sites while safeguarding healthy tissues. Jannesari et al. (2023) proposed an oxygen-enriched ZnO_2_-Ag nano-framework material based on reduced graphene oxide, which enables the controlled generation of oxygen nanobubbles (O_2_NBs) with hydroxyl radicals through pH-responsive catalytic behavior. This study innovatively combined the dual enzymatic activity (CAT/POD) of Ag nanoparticles with the decomposition property of ZnO_2_, which preferentially triggered the generation of hydroxyl radicals by POD activity under acidic conditions and the release of O_2_NBs via CAT activity in neutral/alkaline environments. The growth and rupture of O_2_NBs bubbles can mechanically disrupt the structure of the biological membrane, while the photothermal effect of rGO (under NIR laser irradiation) further accelerates the decomposition of ZnO_2_ and the movement of NBs, thereby enhancing the antibacterial effect.

**FIGURE 7:**
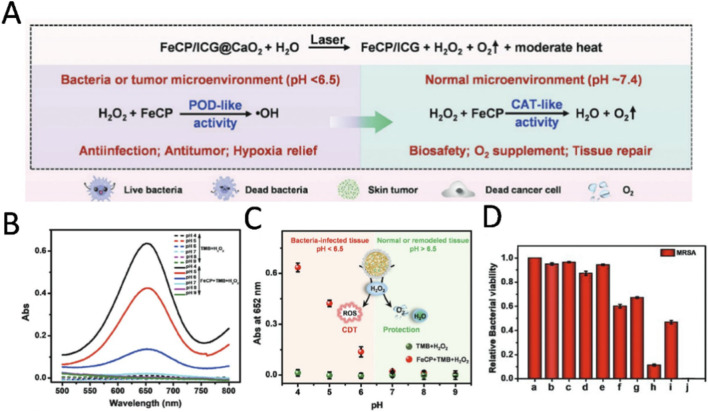
**(A)** Schematic illustration of the potential mechanism of action of FeCP/ICG@CaO_2_ in wound healing of drug-resistant bacterial infections. **(B)** The UV visible absorption spectra and corresponding absorbance values of TMB and H_2_O_2_ mixed solutions with and without FeCP at a wavelength of 652 nm. **(C)** Under acidic conditions (pH 4-6), the addition of FeCP resulted in a significant characteristic absorption of the TMB solution at 652 nm due to the generation of oxidized TMB (oxTMB). **(D)** Quantitative evaluation of bacterial survival rate in agar medium. a–j: PBS; PBS + NIR; FeCP; FeCP + NIR; FeCP/ICG; FeCP/ICG + NIR; FeCP/ICG@CaO_2_ (100 μg·mL^−1^); FeCP/IGC@CaO_2_ + NIR (100 μg·mL^−1^); FeCP/ICG@CaO_2_; FeCP/IGC@CaO_2_ + NIR. Reproduced with permission from reference (Yan et al., 2023).

The physiological concentration of H_2_O_2_ at the site of bacterial infection is merely 50–100 μM. Conventional nanozymes, which rely on endogenous H_2_O_2_ for CDT, struggle to achieve effective levels of ROS necessary for bactericidal activity. Yan et al. (2023) developed an intelligent system (FeCP/ICG@CaO_2_) based on ferricyanide polymer (FeCP) nanozymes, indocyanine green (ICG) and calcium peroxide (CaO_2_) for the treatment of skin tumor wounds infected with drug-resistant bacteria (Figure 7A). The system not only has pH-switchable POD and CAT activities, but also can self-supply H_2_O_2_ and O_2_, and combines with PTT to achieve efficient antibacterial and promote wound healing. When CaO_2_ comes into contact with the moisture in the wound, it decomposes to produce H_2_O_2_ and O_2_. FeCP works like catalase in acidic environments, converting H_2_O_2_ into strong oxidizing hydroxyl radicals that help kill bacteria (Figure 7B). In a neutral environment, FeCP acts like a catalytic enzyme, decomposing H_2_O_2_ to prevent oxidative damage to healthy tissues (Figure 7C). ICG can generate heat under near-infrared light irradiation, which helps FeCP better exert its enzyme like activity and thus improve the therapeutic effect (Figure 7D). In experiments, this nano platform was used to combat drug-resistant bacteria such as MRSA, and its therapeutic effects on MRSA infected wounds and skin tumor wounds were tested in animal models. These features and advantages make FeCP/ICG@CaO_2_ an intelligent system that can overcome the major limitations in treating bacterially infected wounds (e.g., hypoxia, insufficient H_2_O_2_ supply, and adverse side effects) and exhibit satisfactory therapeutic effects on MRSA infected in normal skin wounds and special skin tumor wounds.

### 4.4 Anti-inflammatory action

Oxidative stress, resulting from an imbalance between oxidants and antioxidants, plays a significant role in both acute and chronic inflammation (Conner et al., 1995). ROS, such as superoxide anions, hydroxyl radicals, and H_2_O_2_, are key players in this process (Cuzzocrea et al., 2004). ROS play a significant role in the inflammatory response by activating transcription factors like nuclear factor-kappaB (NF-κB), which in turn regulate the expression of genes involved in inflammation (Pashkow et al., 2008). ROS can initiate lipid peroxidation, inhibit mitochondrial enzymes, and cause DNA damage, leading to cell death and tissue injury, which are central to the pathophysiology of inflammation (Cuzzocrea et al., 2004). Antioxidants control inflammation by neutralizing ROS and reducing oxidative stress, which mitigates cellular damage and modulates inflammatory pathways (Forman and Zhang, 2021). pH-responsive nanozymes offer significant advantages in the treatment of inflammation compared to traditional drugs. These advantages stem from their ability to target inflamed tissues selectively, modulate oxidative stress, and provide controlled drug release in response to the acidic environment typical of inflamed areas.

#### 4.4.1 Simulate antioxidant enzymes

**FIGURE 8:**
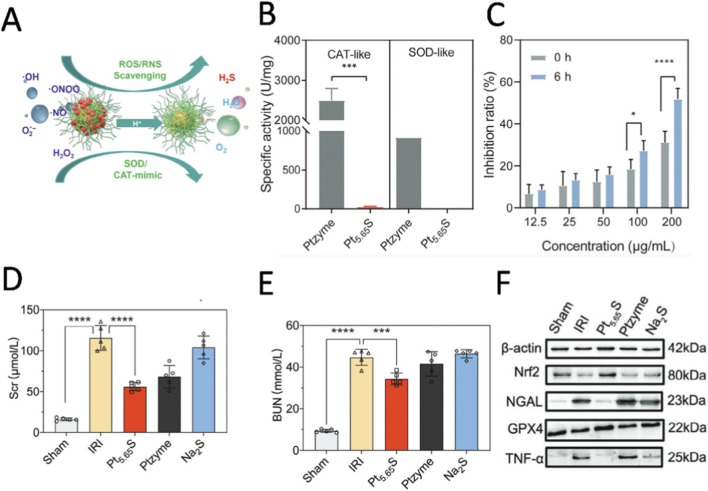
**(A)** Schematic diagram of activity change, antioxidant performance and hydrogen sulfide release process of Pt_5.65_S pre-nanozyme under different pH conditions. **(B)** Measure the CAT/SOD like activity of Pt enzyme and Pt_5.65_S pre nanozyme in a pH 7.4 buffer solution. **(C)** The changes in SOD like activity of Pt_5.65_S nanozyme in pH 6.5 buffer solution after incubation for different times. **(D)** Determination results of serum creatinine (Scr) concentration in each group. **(E)** Determination results of serum urea nitrogen (BUN) concentration in each group. **(F)** Western blot analysis was performed to detect the protein expression levels of NGAL, Nrf2, GPX4, and TNF-α across all experimental groups, followed by quantitative assessment of the protein expression profiles. Reproduced with permission from reference (Jiang et al., 2024).

The core pathological mechanism of acute kidney injury (AKI) is oxidative stress, characterized by the accumulation of excessive ROS; while the inflammatory microenvironment of the kidneys exhibits an acidic pH characteristic, with the interstitial pH at sites of inflammation falling to approximately 6.5, whereas under normal physiological conditions it remains close to 7.4 (Pavlakou et al., 2017). This acidic environment not only exacerbates oxidative stress damage but also provides a potential target for specific therapeutic interventions. In recent years, the application of pH-responsive nanozyme in anti-inflammatory therapy has been further expanded. Jiang et al. (2024) developed a pH-activatable pre-nanozyme, Pt_5.65_S, for hydrogen sulfide (H_2_S) delivery to regulate oxidative stress in AKI (Figure 8A). Under normal physiological conditions (pH 7.4), Pt_5.65_S has low enzyme activity and does not significantly release H_2_S. However, in acidic environments (pH 6.5), mimicking inflammatory sites, the pre-nanozyme activates, releasing H_2_S and transforming into Ptzyme, which exhibits activities resembling superoxide dismutase and catalase (Figures 8B,C). This transformation allows Ptzyme to effectively eliminate excess reactive oxygen species and nitrogen species, alleviating oxidative stress. In the mouse kidney ischemia-reperfusion injury model, Ptzyme effectively reduced serum creatinine and blood urea nitrogen levels, alleviated kidney injury, and improved function (Figures 8D,E). Research has shown that it upregulates Nrf2 and GPX4 expression, inhibits NF - κB activation, and reduces the production of inflammatory factors (Figure 8F). *In vitro* and *in vivo* assessments demonstrated the robust antioxidant activity and biosafety of Pt_5.65_S, indicating its potential as a therapeutic approach for inflammatory diseases such as AKI.

#### 4.4.2 Regulating the immune microenvironment

**FIGURE 9:**
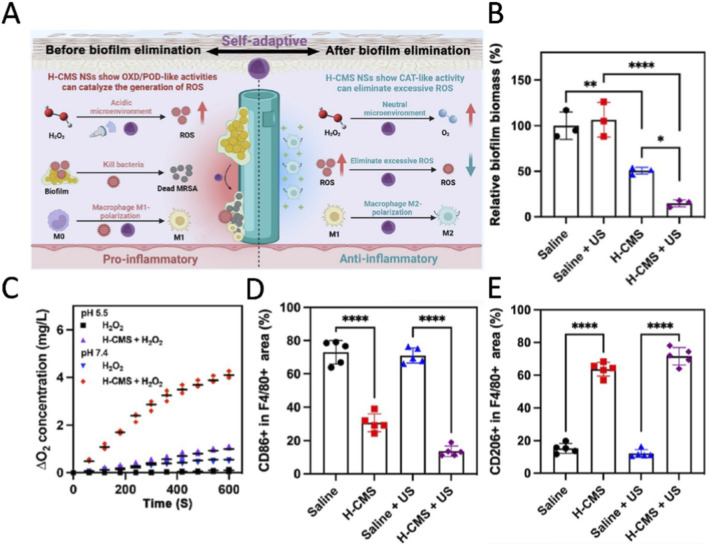
**(A)** Schematic illustration of hollow Cu_2_MoS_4_ nanospheres (H-CMSNSs) exhibiting adaptive antimicrobial membrane properties and immunomodulation for the treatment of implant-associated infections. **(B)** Biomass of methicillin-resistant *Staphylococcus aureus* biofilms growing on implants after 7 days of treatment. **(C)** Measurements of oxygen (O_2_) concentration in hydrogen peroxide (H_2_O_2_) solution after incubation with H-CMS nanosheets showed that the CAT-like activity of HCMSNSs under neutral conditions was significantly higher than the CAT-like activity under acidic conditions. **(D)** The proportion of CD86-positive cells within the F4/80 + region in the peri-implant infected tissue (F4/80, macrophage marker; CD86, M1 marker). **(E)** The proportion of CD206-positive cells within the F4/80-positive region in the peri-implant infected tissues (CD206, M2 marker). Reproduced with permission from reference (Yang et al., 2023).

The treatment of implant infections faces two core challenges: first, the antibiotic tolerance mediated by biofilms, and second, the balanced regulation of inflammatory responses at different stages of infection (Costerton et al., 1999; Lukoyanychev et al., 2022). In the state of biofilm infection, bacteria induce local microenvironment acidification (pH 4.4) through anaerobic metabolism and the respiration of immune cells; conversely, once the infection is cleared, the tissue microenvironment gradually returns to neutrality (pH 7.4) (Behbahani et al., 2022). Biofilm infections also suppress the pro-inflammatory responses of macrophages, leading to immune evasion (Alboslemy et al., 2019). Traditional therapies, if they continue to activate inflammation after biofilm clearance, may result in secondary tissue damage (Costerton et al., 1999). In contrast, pH-responsive nanozymes achieve a comprehensive adaptive therapy encompassing bactericidal action, immune modulation, and tissue repair by sensing the pH changes in the microenvironment of implant infections. Yang et al. (2023) subsequently developed multifunctional hollow Cu_2_MoS_4_ nanospheres (H-CMS NSs) designed for the self-adaptive treatment of implant infections (Figure 9A). In the acidic environment characteristic of biofilm infections, H-CMS NSs display oxidase and POD activities that catalyze the generation of ROS, facilitating direct bacterial killing and polarizing macrophages towards a pro-inflammatory phenotype. Moreover, the therapeutic effect of H-CMS NSs is enhanced under ultrasound irradiation, further boosting their antibacterial and POD-like properties (Figure 9B). As the biofilm is eliminated, the surrounding tissue environment transitions from acidic to neutral, allowing H-CMS NSs to shift their activity to mimic CAT (Figure 9C). This transition helps to eliminate excessive ROS, subsequently polarizing macrophages to an anti-inflammatory phenotype, which promotes tissue healing (Figures 9D,E). This innovative approach offers significant advantages in the context of implant infections by providing a dynamic regulatory mechanism for both antibiofilm action and immune modulation, adapting to the evolving pathological microenvironments throughout the treatment process.

### 4.5 Other diseases

**FIGURE 10:**
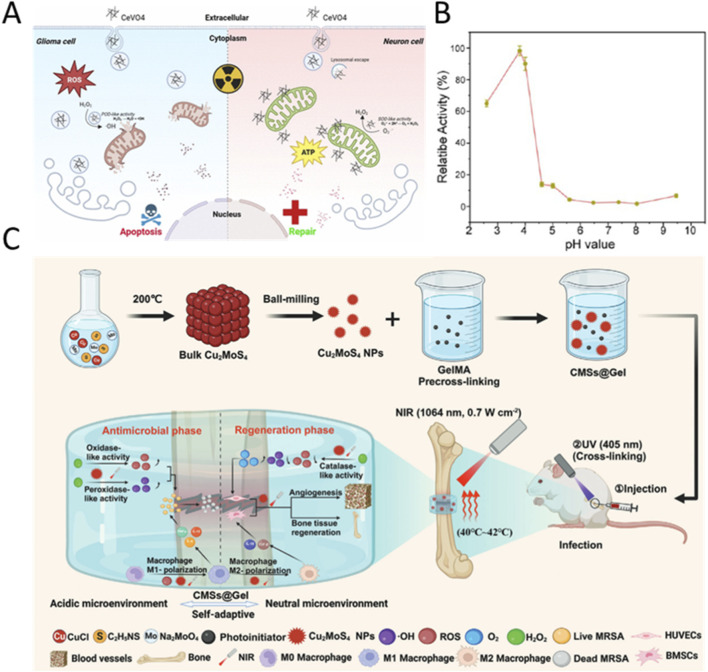
**(A)** Schematic diagram of the catalytic therapeutic mechanism of CeVO_4_. Under the action of ionizing radiation, locally injected CeVO_4_ exhibits pH-dependence, which enables it to target and kill glioma cells while protecting neurons. **(B)** Plot of the peroxidase-like activity of CeVO_4_ as a function of pH. Reproduced with permission from reference (Han et al., 2024). **(C)** Schematic diagram of *in vitro* synthesis and *in vivo* mechanism of action of CMSs@Gel. Reproduced with permission from reference (Xiu et al., 2025).

In recent years, nanobiotechnology has made rapid progress, and researchers are actively working to expand the application of novel pH-responsive nanozymes in the clinic to encompass the treatment of a wider range of diseases (Zhu et al., 2025). The treatment of radiation-induced brain injury following glioblastoma multiforme presents a dual challenge: it is essential to eradicate residual tumor cells while simultaneously safeguarding normal neurons from radiation-induced oxidative damage. Han et al. (2024) developed a CeVO_4_ nanozyme with pH-responsive dual enzyme activity, which reveals its dual functional mechanism in glioblastoma treatment and radiation brain injury protection (Figure 10A). It was found that the nanozyme exerted peroxidase-like activity in the lysosomal acidic environment of glioblastoma cells, and enhanced the tumor cell killing effect through the generation of reactive oxygen species in synergy with radiotherapy (Figure 10B), and meanwhile it was confirmed by *in vivo* experiments that it could significantly inhibit the growth of grafted tumors *in situ* and prolong the survival period of mice with loaded tumors. In neuronal cells, CeVO_4_ escapes from lysosomes and targets mitochondria through the fast endocytosis pathway, and exhibits superoxide dismutase-like activity in a near-neutral environment, which effectively scavenges radiation-induced mitochondrial superoxide radicals, restores ATP levels and mitochondrial membrane potential, and thus reduces apoptosis in neurons. This work achieves for the first time the precise regulation of the function of a single nanozyme in tumor cells and normal neuronal cells, and provides a new paradigm for the development of synergistic strategies for targeted therapy and neuroprotection of brain tumors.

The microenvironment at the site of fracture infection exhibits significant pH phase characteristics: during the acute infection phase, the local environment becomes acidic (pH 5.5) due to bacterial acid metabolism and the enhanced glycolysis of inflammatory cells (Sun et al., 2024). As the infection is controlled and the secretion of osteogenic and angiogenic factors increases, the microenvironment gradually transitions to a neutral state (pH 7.4), marking the initiation of the repair phase (Spector et al., 2001). pH-responsive nanozymes, due to their ability for “smart switching,” can effectively combat infection during the bactericidal phase and precisely mitigate inflammation to promote healing during the repair phase, thereby avoiding the interference of single-function nanozymes in the treatment process. Xiu et al. (2025) developed a photo-thermo-responsive hydrogel (CMSs@Gel) based on the composite of copper-molybdenum sulphide nanomaterials (CMSs) with gelatin methacrylate, which was synthesized by ball milling method CMSs and encapsulated in a hydrogel (Figure 10C). In the acidic microenvironment at the early stage of infection, its peroxidase and oxidase activities are enhanced, which destroys the structure of bacterial membranes through the generation of ROS and hydroxyl radicals, and activates the M1 polarization of macrophages to enhance the inflammation response; in the late neutral microenvironment, its catalase activity was significantly elevated, scavenging excess ROS and promoting macrophage transformation to M2 phenotype, while promoting osteogenic differentiation gene (RUNX-2, OCN) expression and angiogenic factor (VEGF, CD31) secretion through the photothermal effect (42 °C). The material demonstrated long-lasting slow-release properties and bone repair efficacy in in vivo experiments, which elevated the bone volume fraction of the fracture site to 85% of normal bone, and provided a novel multifunctional material platform for the treatment of fracture-associated infections.

## 5 Challenges and strategies for clinical translation

Research on the clinical translation challenges of pH-responsive nanozymes has emerged as a critical area of inquiry due to their promising therapeutic efficacy in treating tumors, inflammation, and infections. Since the initial discovery of enzyme-like activities in nanomaterials, the field has evolved rapidly, with early studies focusing on catalytic mechanisms and later expanding to multifunctional applications in tumor microenvironment-responsive imaging and therapy (Wang H. et al., 2024). Despite these advances, the translation of nanozyme-based therapies from animal models to clinical settings remains limited. The pH microenvironment in organisms is highly heterogeneous and dynamic, which leads to significant differences in pH conditions between animal models and human physiological tissues, thus severely limiting the clinical translation process of ph-responsive nanozymes (Nair et al., 2024). Many promising preclinical results have not been replicated in clinical trials, highlighting gaps in model predictiveness and the need for improved translational frameworks (Yang et al., 2025).

To address these limitations, researchers are adapting pH-responsive nanozymes to dynamic physiological environments by modulating local pH to match their optimal activity range, where precise regulation of acid-base properties at active sites is critical for advancing their catalytic efficiency and specificity in biomedical applications. For example, the integration of polyelectrolytes into nanozyme architectures enables localized pH modulation, thereby significantly enhancing enzymatic activity at physiological pH (Li T. et al., 2024). Li et al. achieved precise regulation of microenvironmental pH at the active site by confining poly (acrylic acid) (PAA) or poly (ethyleneimine) (PEI) in the channels or cavities of MOF nanozymes. The confinement of PAA lowers its microenvironmental pH, enabling it to perform its best activity at pH 7.4 and to solve pH mismatch in cascade systems coupled with acid-denatured oxidases. Conversely, the confinement of PEI increases the microenvironmental pH, leading to the enhanced hydrolase-mimicking activity. Another approach is to activate the nanozymes *in situ* by using glucose oxidase to locally lower the pH and increase the H_2_O_2_ concentration (Chen L. et al., 2021). Specifically, systems like the “APGH” nano-capsule, comprising aptamer-functionalized nanozymes, glucose oxidase, and hyaluronic acid, demonstrate this principle by utilizing glucose oxidation to artificially tune the local pH down, thereby creating an acidic niche optimal for nanozyme activity where it otherwise would not exist. Such precise control over local acid-base properties not only enhances catalytic efficiency but also minimizes off-target effects by restricting activity to specific pathological microenvironments, offering a promising pathway toward clinically viable nanozyme therapeutics with improved safety profiles and therapeutic indices.

With the rapid development of nanotechnology towards precision therapy, more effective solutions have emerged. Researchers are striving to develop nanozymes that respond to various parameters such as pH, temperature, redox potential and inflammatory markers, and will combine external stimuli such as light and heat, magnetic field or ultrasound to develop multimodal control system similar to pH + temperature + redox, which can be accurately manipulated through spatiotemporal control strategies to enhance lesion localization. This system can precisely manipulate the enzyme-like activity through spatio-temporal control strategy and enhance the ability of lesion localization. In addition, researchers can explore the synergistic mechanism of different valence elements (e.g., Cu^2+^/Mn^3+^/Mn^4+^) in the multi-metallic composite nanozymes, and enhance the sensitivity of the pH response by precisely regulating the ratio of the elements and the crystal structure, in order to improve the dynamic adaptability to the micro-acidic environment of tumors or the neutral environment of wound infections (Hu et al., 2025).

## 6 Conclusion and perspectives

pH-responsive nanozymes combine the physicochemical properties of nanomaterials with the catalytic activity of natural enzymes, and are able to precisely regulate the catalytic activity through changes in microenvironmental pH, showing promise in addressing unmet clinical needs in areas such as infection therapy, tumor treatment, and tissue repair. Through innovative design, it effectively solves the problems of traditional therapeutic means, such as difficulty in precisely acting on pathological sites and easy to produce off-target toxicity, and provides a new way of disease treatment with higher efficiency and lower toxicity.

Various pH-responsive nanozymes achieve intelligent catalytic activity modulation in different pH environments through their unique material properties, such as the surface charge effect of noble metals, the valence change of metal oxides, the structural change of sulfides, the structural stability of MOFs, and the change of surface functional groups of carbon dots. To realize the precise activation of pH-responsive nanozymes, surface modification, compositional doping, and core-shell structural engineering strategies can be used to intelligently regulate them at the interfacial property, intrinsic electronic structure, and spatial integration levels, respectively, so as to construct targeted diagnostic and therapeutic platforms with even better performance. The pH-responsive nanozymes utilize the characteristic pH conditions of pathological microenvironments such as cancer, infection and inflammation, and the targeted activation at the site of these foci exerts functions such as antibacterial, anti-inflammatory, scavenging or generating reactive oxygen species, promoting tissue repair, etc., and exhibits great therapeutic potential. In addition, it has application prospects in other disease areas such as glioblastoma and bone repair.

pH-responsive nanozymes are facing multiple serious challenges from laboratory to clinical applications, such as biosafety, scale-up production, response accuracy, evaluation system and regulatory compliance, etc. In the future, these obstacles can be systematically solved through cross-disciplinary collaborative innovation in order to ultimately realize their clinical translational value. Integration of computational tools, such as density functional theory and machine learning, can facilitate the rational design of nano-enzymatic structures, optimize the adsorption capacity of substrates such as O_2_ and H_2_O_2_, and further amplify pH-dependent activity (Chen Y et al., 2023; Wu et al., 2025). The integration of diagnostic modalities (including fluorescence/photo-acoustic imaging or electrochemical biosensors) into the therapeutic platform is expected to enable dynamic feedback of pH and reactive oxygen species levels in the lesion area to optimize the therapeutic window (Yu et al., 2023). The combination of materials science, computational chemistry, microbiology and clinical medicine will provide new perspectives and approaches to address existing problems. Through continuous exploration and innovation, pH-responsive nanozymes are expected to provide more effective therapeutic options for a wide range of diseases, such as infections, tumors, and inflammatory bowel diseases, and ultimately lead to more precise and personalized medical treatments.
